# The Micrococcus of Dengue

**Published:** 1885-11

**Authors:** 


### THE MICROCOCCUS OF DENGUE.

### AN IMPORTANT DISCOVERY BY A TEXAS PHYSICIAN.

The recent epidemic of dengue has not been, it is hoped, without its lessons. While it cannot be claimed positively, that anything of practical value has been learned as to the etiology or prophylaxis of this disease, enough has been ascertained regarding its pathology and clinical history to lead to the belief that it is a disease of the blood—produced by an organism,—a *contagium vivum*, and that it is both contagious and infectious.

Dr. J. W. McLaughlin, President of the Texas Microscopic Association, instituted a series of examinations of the blood and the urine of dengue patients taken in every stage of the disease. The blood was extracted with a sterilized hypodermic needle with every precaution against atmospheric contamination, and was placed on sterilized discs. These he dried, and treated, some with glacial acetic acid, and some with *liquor potassae*, and stained,—some with analine, and some by the new method of Gram of Copenhagen. Upon examination, Dr. M. found myriads of *organisms* unlike any that have ever been seen or described, heretofore, in any fever, so far as he has been able to ascertain, and the doctor thinks he has good reason for believing them to be the *true germs* of dengue. It is well known that by the methods of staining employed, nothing retains the dye but an organism,—while none of the tissues or elements of the body can withstand the acetic acid or the caustic potash, which, however, have no destructive effect on these singular organized atoms. Under the microscope large numbers of them are seen.

They are exceedingly small, circular or oval bodies of a reddish hue, encased in a darker outline, and are about the twentieth to the thirtieth of the size of an ordinary red blood corpuscle—i. e., a blood corpuscle is capable of containing from fifteen to thirty of them. And here is another singular fact—they are seen occupying the red blood cells in numbers, something not before observed, according to Friedlander, who avers that in no disease and in no condition of life, have *micrococci* or other organisms been known to enter and occupy the blood cell. Dr. M. finds the *micrococci* also in groups, technically called *zooglœa*, and in garlands; and, in the urine, formed into distinct tube casts.

It is proper to state that these same bodies were found abundently in the atmosphere also, and the doubt which attaches to all such investigations arises here, as to whether they are the cause, or one of the products of the disease. The Doctor has isolated what he believes to be the true dengue germ, and has succeeded in reproducing them in Miguel's culture of Irish moss and beef broth. He will attenuate these and try the effects of inoculation.

Who knows but what a Texas physician may have placed within the grasp of science the preventive of this dread disease, which, while non-fatal, is nevertheless very distressing; and which, in addition to causing suffering, loss of time, and pecuniary loss, occasions also much profanity? For we venture that most of the victims speak of the disease, and will do so to their dying day, with a prefix more forcible and expressive than elegant.

P. S.—Since writing the above we have examined the Doctor's collection. He showed us a beautiful specimen of the *mycelium* with its *conidia*, and myriads of its spores or *micrococci*. There can be but little doubt that Dr. McL. has made an important find—one which we believe will prove a valuable addition to our knowledge of the etiology of our Southern fevers.

